# Sustainable memristors from shiitake mycelium for high-frequency bioelectronics

**DOI:** 10.1371/journal.pone.0328965

**Published:** 2025-10-10

**Authors:** John LaRocco, Qudsia Tahmina, Ruben Petreaca, John Simonis, Justin Hill

**Affiliations:** 1 Psychiatry and Behavioral Health, Wexner Medical Center, Ohio State University, Columbus, Ohio, United States of America; 2 College of Engineering, Ohio State University, Columbus, Ohio, United States of America; 3 College of Arts and Sciences, Ohio State University, Columbus, Ohio, United States of America; Shenzhen University, HONG KONG

## Abstract

Neuromorphic computing, inspired by the structure of the brain, offers advantages in parallel processing, memory storage, and energy efficiency. However, current semiconductor-based neuromorphic chips require rare-earth materials and costly fabrication processes, whereas neural organoids need complex bioreactor maintenance. In this study, we explored shiitake (*Lentinula edodes*) fungi as a robust, sustainable alternative, exploiting its adaptive electrical signaling, which is akin to neuronal spiking. We demonstrate fungal computing via mycelial networks interfaced with electrodes, showing that fungal memristors can be grown, trained, and preserved through dehydration, retaining functionality at frequencies up to 5.85 kHz, with an accuracy of 90 ± 1%. Notably, shiitake has exhibited radiation resistance, suggesting its viability for aerospace applications. Our findings show that fungal computers can provide scalable, eco-friendly platforms for neuromorphic tasks, bridging bioelectronics and unconventional computing.

## Background

### Overview

The development of neuromorphic hardware relies on memristive devices capable of emulating synaptic behavior, with potential applications in energy-efficient computing and artificial intelligence^1^. Recent work has explored natural, biodegradable substrates as sustainable alternatives to conventional inorganic memristors [1]. In this study, we investigated the potential of the edible fungus *Lentinula edodes* (shiitake mushroom) as a platform for memristor fabrication. By examining the electrical response of mushroom-derived materials under repeated voltage cycling, we explored stable memristive switching behavior, retention, and endurance. Shiitake-based devices not only demonstrate reproducible memory effects, but also highlight the potential for scalable, low-cost, and environmentally friendly neuromorphic components.

### Memristors

Memristor devices offer substantial advantages in robotic, industrial, and transport applications due to their unique electrical properties and ability to mimic neural functions. They can enhance various control systems, facilitate efficient information processing, and ultimately improve the overall performance of autonomous systems.

One of the key strengths of memristors is their capacity for efficient and self-adaptive in situ learning, which is critical for applications in robotics and autonomous vehicles. In memristor-based neural networks, the devices can adjust their resistance based on previous inputs, allowing for a form of analog learning that closely resembles the synaptic behavior in biological systems [1]. This capability enables robots and autonomous vehicles to learn from their environment and adapt in real time, enhancing their ability to navigate complex situations effectively. It has been found that such systems can achieve low-latency responses, which are essential for high-speed decision-making in dynamic environments [2].

Memristors also have the advantage of integrating memory and processing capabilities into a single device, enabling a simplified architecture for autonomous control systems [3]. For instance, in autonomous vehicles, trajectory-tracking and path-following tasks can be performed using memristor-based controllers that allow for rapid calculations and real-time adjustments to control variables [4]. This integration, especially with parallelization, helps to address the challenges posed by separate memory and processing units, which can lead to delays and increased power consumption in traditional control systems [4].

Additionally, the resilience of memristor devices against environmental changes, and their ability to operate under varying conditions, make them particularly suitable for autonomous applications, such as spacecraft electronics or vehicles operating in unpredictable road environments [4]. This is complemented by the precision in control that memristor-based systems can offer, which is significant for maintaining stability and performance while following desired trajectories [5].

Moreover, the low power consumption of memristors is particularly beneficial in robotics and autonomous vehicles, where energy efficiency is paramount. Hybrid analog–digital memristor systems can minimize power usage during processing without sacrificing responsiveness, which can prolong operational times by reducing the frequency at which recharging or battery replacement is required, enhancing the feasibility of deploying such systems in mobile applications [2].

Ultimately, the potential of memristors to emulate human-like decision-making and learning processes could be exploited to endow robotic systems and autonomous vehicles with functionalities not found in conventional control systems. The ability of memristors to perform complex computations efficiently, learn adaptively, and integrate both memory and processing into a unified approach make them a cornerstone technology for the future development of intelligent autonomous systems. However, the production of memristors often requires rare-earth minerals and expensive semiconductor foundries.

### Fungal electronics

Fungi possess innate abilities to adapt to various environmental conditions and efficiently process information through their interconnected network of hyphae. These characteristics make fungi an ideal candidate for developing sustainable computing systems from. Our aim was to design and implement a novel fungal memristor-based computing architecture that could significantly reduce energy consumption and minimize electronic waste. We approached this using substantially simpler bioreactors and nutrient cultures than those required for conventional neurons and neural organoids. The unique advantages of fungal memristors stem from the biological properties of fungal materials, which distinguish them from typical inorganic or polymer alternatives [6,7].

First, one of the main benefits of fungal memristors is their environmentally sustainable and biodegradable nature. Conventional memristors often contain transition metal oxides or silicon-based structures, the production or disposal of which can pose environmental challenges [6,7]. By contrast, fungal materials are derived from organic biomass, making them both sustainable and significantly less harmful to the environment. This aligns with increasing efforts toward developing greener electronic materials, as highlighted in previous work emphasizing the importance of sustainability in technology development [8].

Second, fungal memristors exhibit remarkable adaptability in their electrical properties. The structural composition of fungal materials often allows for a range of conductive pathways that can form dynamically under the influence of electrical stimuli, similar to the conductive filaments formed in conventional memristors [9,10]. This adaptability can lead to enhanced performance in neuromorphic applications through the facilitation of variable resistance states that mimic synaptic behaviors more closely than traditional memristive materials, which often have static crystalline structures that can lead to variability problems or performance limitations at the nanoscale [11].

Furthermore, fungal memristors may consume less power than traditional materials due to their unique electrochemical properties. It has been claimed that some organic materials, including those derived from fungi, can operate effectively at lower voltages while maintaining stable switching characteristics––a trait that is crucial for developing energy-efficient devices for portable electronics and Internet of Things applications [12]. This can significantly extend battery life and reduce energy costs in processing and memory applications, which have become focal points in the research into neuromorphic systems [13].

Finally, the natural composition and multicellularity of fungal materials can lead to more naturalistic models for neural networks. Because these materials are subject to biological processes, they may inherently incorporate characteristics that resemble biological neuronal networks, including plasticity and memory capabilities that could evolve with usage. This biological mimicry could strengthen the development of more advanced artificial neural networks, enabling applications such as adaptive learning systems and intelligent sensor networks [14].

### Fungus types

The potential use of common food mushrooms, such as shiitake and button mushrooms (*Agaricus bisporus*), as organic memristors is an emerging area of research that exploits the unique properties of these fungi [6,7,13]. Memristors, which are non-volatile memory devices that retain information even without power, can benefit from the porous structures and electrical properties of the organic materials derived from mushrooms.

Shiitake mushrooms have been shown to possess a hierarchically porous carbon structure when activated. This porous structure can enhance the electrochemical performance of devices, making them suitable candidates for use in energy storage systems, including supercapacitors and, potentially, memristors [15]. Highly conductive carbon materials have been created from shiitake, suggesting that these materials could be engineered to exhibit memristive behavior [16]. Shiitake-derived carbon is a sustainable alternative to traditional materials and can enhance the performance of electronic devices due to its unique structural properties.

Button mushrooms have also shown significant potential in this context. Research has indicated that their porosity can be exploited to create materials with large surface areas, which are essential for the development of efficient electronic components [17]. The synthesis of carbon composites from button mushrooms has been explored, revealing their ability to function effectively in energy storage applications [17]. Furthermore, the integration of button mushrooms into electronic systems has been investigated, demonstrating their potential as substrates for electronic devices [18].

In addition to their structural properties, the unique biological characteristics of fungi, including their ability to interact with various chemical compounds, can be harnessed to develop novel sensing technologies. For instance, electronic noses have been developed that use mushroom extracts to detect volatile compounds. These could be adapted for use in electronic devices that require environmental-sensing capabilities [19,20]. This intersection of biology and electronics opens new avenues for creating multifunctional devices that incorporate the sensory capabilities of mushrooms.

### Radiation, resistance, and resilience

The radiation resistance of shiitake mushrooms has been studied primarily in terms of their ability to withstand and possibly derive benefits from exposure to ionizing radiation. This resistance can be attributed to several biochemical and physiological attributes. A possible factor is lentinan, a polysaccharide found in the cell walls of shiitake. Lentinan provides structural integrity and exhibits immunomodulatory effects that may enhance the mushroom’s ability to respond to environmental stresses, including radiation exposure. Although some research has suggested that lentinan possesses properties that may help mitigate oxidative stress [21], there have been limited studies directly linking lentinan to radiation resistance in shiitake mushrooms.

Shiitake mushrooms have also shown a notable ability to adapt to their environmental conditions, including variable radiation levels. Studies involving fungi in space research have indicated that certain taxa can enhance their survival through morphological changes or increased melanin production in response to radiation [22]. This radiation resistance implies a suitability of fungal electronics for aerospace applications, where cosmic rays and ambient radiation can interfere with conventional electronics. Fungi’s physical flexibility and low energy requirements would also be advantageous relative to conventional solutions [18,19]. These studies have not specifically addressed shiitake, but the general adaptability observed in fungi suggests that this species could respond similarly to such conditions.

Another example of the resilience of shiitake mushrooms is their ability to maintain their nutritional and bioactive qualities after irradiation. For example, they retain essential nutrients and bioactive compounds even after exposure to ultraviolet radiation [23]. The high content of ergosterol, a precursor to vitamin D, found in shiitake mushrooms, reinforces their potential for beneficial outcomes following exposure to radiation because this compound can be converted into vitamin D_2_ when subjected to ultraviolet light [24].

Lastly, shiitake mushrooms could be considered in the development of dietary supplements or functional foods that could serve a broader purpose in radioprotection. Their multirole efficacy as a food source and electrical component emphasizes a sustainable approach to utilizing biological entities that can withstand environmental stresses, including radiation. This is especially relevant in aerospace and exploration contexts, where promoting health in astronauts could reduce the risks associated with their increased radiation exposure during missions [22]. Also, shiitake mushrooms can withstand environmental stresses, including radiation, while remaining safe for human consumption.

In summary, the radiation resistance of shiitake mushrooms is linked to the presence of protective compounds, such as lentinan, and their ability to adapt morphologically. These factors have contributed to our understanding of their survival strategies and are suggestive of potential applications in areas where radiation exposure is a significant concern, such as aerospace and radiation sensing. By culturing and evaluating the memristive properties of shiitake mushrooms, we can determine their suitability for use as sustainable, low-cost bioelectronics.

## Methods

### Summary

Testing the memristive behavior of shiitake mycelium involved several steps, the first being culturing the fungi, and then preparing the samples by drying and rehydrating them. Following this, the most successfully cultivated samples were electrically characterized using a test circuit. Additionally, a special circuit was constructed for further evaluating the feasibility of using mycelium for violate memory.

### Hyphal cultivation

Due to the financial and environmental constraints of this project, all four evaluated memristors fabricated for our experiments were composed exclusively of low-cost, organic materials. Based on previous research, we identified materials such as biocompatible composites [25,26] as viable candidates for device construction and programming due to their biodegradability and compatibility with fungal growth.

The initial phase of experimentation focused on the cultivation of fungal hyphae in the selected organic growth media. Nine samples were prepared in standard polycarbonate Petri dishes. The growth conditions were carefully maintained to promote optimal fungal development, with a controlled temperature range of 20–22°C, a relative humidity of 70%, and mixed light exposure to replicate natural terrestrial conditions. The nutrient substrate consisted of a mixture of farro seed, wheat germ, and hay, selected for their organic compositions and ability to support robust fungal growth. Each sample was inoculated with spores or mycelial plugs of shiitake.

**Fig 1:**
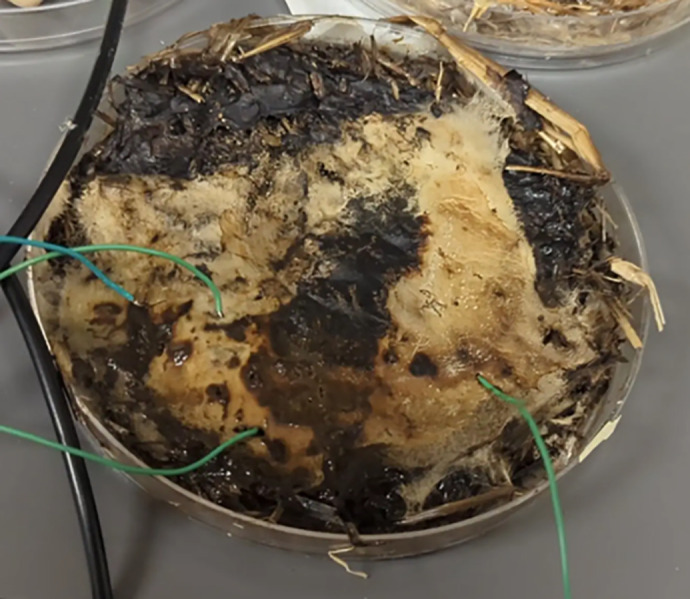
Fungal sample with probe points. Each sample grew a mycelial network that was connected to conventional electronics.

The samples (e.g., see Fig 1) were observed and documented biweekly to track their growth consistency and morphological development. Observations including hyphal density, surface coverage, and color changes were recorded in a structured laboratory logbook. In addition to these visual inspections, a brief scratch test was performed to track the progress of the mycelium throughout the substrate. The log included timestamps and qualitative notes, enabling consistent comparison across samples and time points.

### Drying and rehydration process

Once full hyphal coverage and structural maturation were achieved (i.e., when the Petri dish was covered), the samples were transitioned to the drying phase. The Petri dishes were left in a well-ventilated area under direct sunlight at room temperature for approximately seven days to ensure uniform dehydration. The samples were rotated periodically to avoid uneven hardening. As previously reported, this process transformed the fungal matrix into a rigid, disk-like structure while retaining its overall shape and connectivity [26,27].

Prior to testing, the samples were rehydrated using a fine mist of aerosolized deionized water. The rehydration was conducted using a standard commercial spray bottle, held within a distance of 10 cm from each sample. This brief rehydration step restored the required level of conductivity without introducing bulk moisture that could have altered their mechanical integrity.

### Electrical characterization

Electrical testing protocols were designed based on theoretical memristors [6,7]. An alternating current was applied to each sample, and the corresponding current–voltage (I–V) characteristics were measured using a digital oscilloscope. As established in previous works, the test setup used a voltage divider to model multiple memristors in the same circuit [6,7].

**Fig 2:**
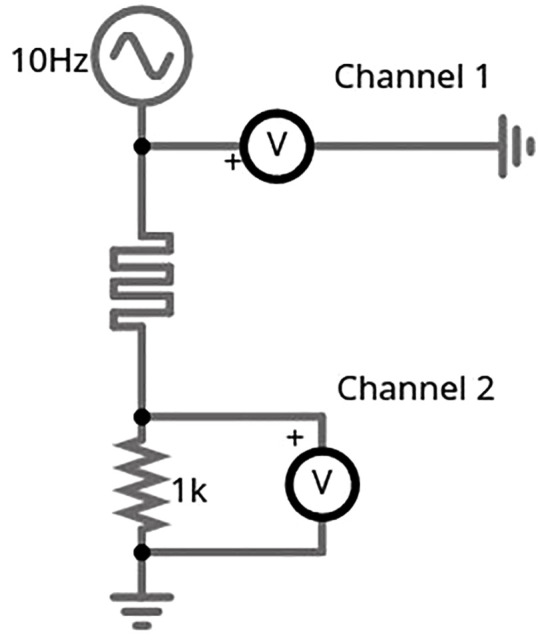
Example theoretical test circuit. The test circuit evaluated the memristive properties of each sample.

To extract accurate current values, a known shunt resistor was placed in series with each sample. As shown in Fig 2, voltage readings were captured across both the sample and the resistor, with Channel 1 of the oscilloscope measuring the input voltage and Channel 2 capturing the voltage drop across the shunt resistor. The current values were then calculated using Kirchhoff’s current law, allowing derivation of the I–V characteristics from the voltage differentials. All waveform data were exported in comma-separated values (CSV) format for subsequent digital analysis and visualization.

To thoroughly investigate the memristive behavior of the four samples using mycelium coverage density, voltage sweeps were conducted using both square and sinusoidal waveforms. The square waves were employed to detect sharp threshold-based resistance changes, whereas the sinusoidal inputs provided insights into the more subtle, continuous mem-fractive behaviors. This dual approach enabled the identification of hysteresis loops in the I–V curves––a key signature of memristor functionality.

A square wave was used first, with the peak-to-peak voltage starting at 200 mV_pp_ and increasing. If a sinusoidal wave form exhibited more promising results, a broader range of frequencies was explored. The frequencies and voltages used in the initial tests for memristive properties are detailed in Table 1.

**Table 1:** Test parameters for memristive testing of the fungal samples.

Test	Voltage (V_pp_)	Frequency (Hz)	Wave
1	0.2	100	Square
2	0.2	200	Square
3	20	200	Square
4	1	200	Square
5	1	200	Sine
6	1	200	Sine
7	1	25	Sine
8	1	50	Sine
9	1	10	Sine
10	5	10	Sine

Accuracy and error were calculated based on how many reads agreed with the analog threshold, the number of malformed readings, timing jitter, recording instability, and port delays [28].


Accuracy (Acc) = 100 * C/N (1)

The accuracy was calculated using Equation 1, where accuracy is a percentage converted from product of correct samples *C* over the total number of samples *N*. The standard error *SE* was calculated for each case, as shown in Equation 2.


$$SE=\sqrt{\frac{Acc(\text{1}-Acc)}{N}}$$ (2)

A simulated ideal memristive curve was compared against each experimental result, where the statistical distance *d* was calculated between both curves [28]. The distance was used to compute memristive accuracy $Acc_{mem}$ at a particular frequency, as shown in Equation 3.


$$Acc_{mem}=(\text{1}-d)*\text{1}\text{0}\text{0}\;$$ (3)

### Volatile memory testing

**Fig 3:**
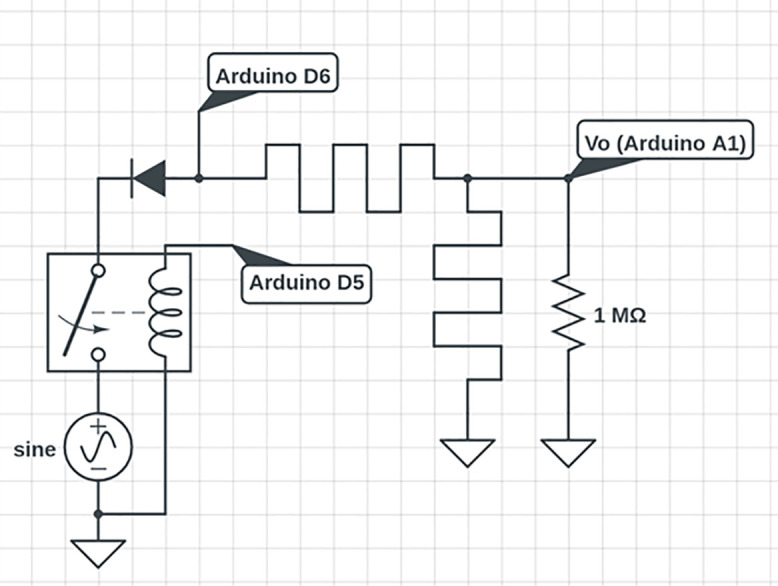
Theoretical volatile memory circuit. The samples were evaluated using this model.

In the event that the fungal samples exhibited memristive behavior, a specialized electronic circuit was designed and implemented to investigate the volatile memory characteristics of two fungal samples in series. The test circuit was a voltage divider with memory. The test involved setting an arbitrary analog voltage value to represent a high value, and below that threshold was a low value. The frequency range started at 200 Hz and concluded at 5.85 kHz. Similarly to previous work, Fig 3 shows the configuration and layout of this testing circuit [6].

**Fig 4:**
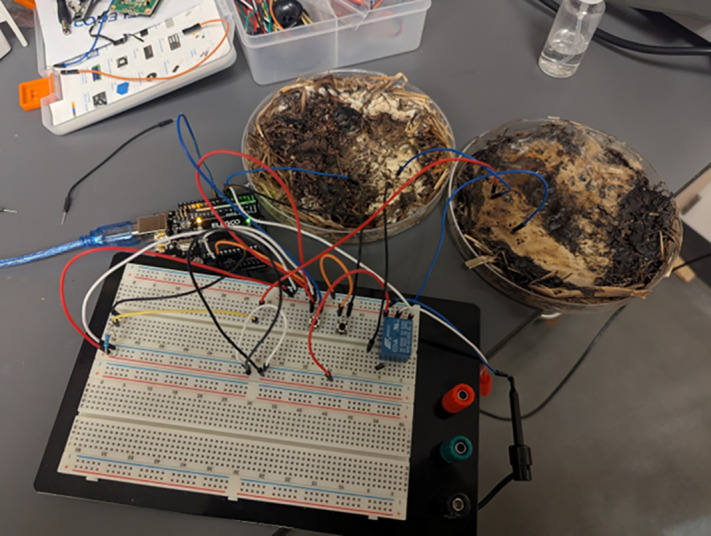
Wired samples. The volatile memory circuit was implemented using fungal memristors.

Comparably to previous work in memristive computing, the volatile memory circuit employed an Arduino UNO microcontroller development board and a voltage divider consisting of two memristive elements [6,7]. Given the polarized nature of memristors, the circuit was designed to allow a voltage of opposite polarity to that used during read operations to be set. Both voltages used were approximately 5 V. The Arduino UNO cyclically applied a high signal to a relay containing a half-rectified sine wave through one of its digital output pins when reading the memristor bridge, thereby charging the divider. This process induced an asymmetry in resistance, with the memristor closest to the input experiencing a reduction in resistance, while the output-side memristor exhibited an increase. The voltage across the divider was subsequently read using an analog input pin, and another digital pin was used to run 5 V across the divider. The Arduino interpreted the stored state as “on” only when the measured voltage exceeded a predefined threshold, effectively enabling volatile memory detection based on the transient resistance states of the memristors. Ten tests were repeated on each of the four samples. The physical implementation of this circuit is shown in Fig 4.

The memristor voltage divider was tested by applying a 5 V_pp_ sinusoidal signal to the memristors for approximately 0.01–0.1 ms. This signal was delivered via a relay triggered by digital pin 6 of the Arduino UNO. Following this brief stimulation period, the sinusoidal input was disabled, and digital pin 5 was activated to initiate the read phase. Analog voltage measurements were then acquired through the A1 analog input pin. To minimize the effects of floating voltages, a 1 MΩ pull-down resistor was connected to this pin. Voltage readings were recorded for approximately 0.1–0.10 ms before the cycle repeated, allowing for rapid and continuous testing of the memristive behavior.

The measurements were transmitted over a serial communication interface at a baud rate of 57,600 and were captured as raw text files for analysis. The data were post-processed and visualized using a custom Python script based on the matplotlib library, enabling clear identification of memory retention patterns and resistance state changes across successive cycles.

### Hypothesis

The general testing setup, based on that used in the literature, is able to indicate memristive behavior in fungal samples. If present, this behavior would manifest as a characteristic pinched hysteresis loop in the I–V curves, typically intersecting at or near the origin––a well-established signature of memristive systems [6,7]. We hypothesized that such a response would emerge under specific combinations of voltage amplitude and input frequency. Where memristive behavior was indicated, volatile memory tests were conducted.

## Results

### Overview

The fungal memristors were tested across a range of voltages, waveforms, and frequencies. Below, we first detail the test inputs used to explore the memristive properties and generate I–V curves. Then we present the voltage and frequency (graphical) test results, followed by the volatile memory test results. Each figure represents the averaged, smoothed results across the samples.

### Voltage testing

The first five tests were conducted to determine which voltage amplitude produced the most favorable memristive response. These initial trials revealed that a 1 V_pp_ signal yielded the most consistent and measurable results. As outlined in the Methods section, the first four of these tests were performed using a square wave input.

### Frequency testing

After identifying 1 V_pp_ as the optimal input voltage during the initial square wave tests (Tests 1–4), the waveform was switched to a sine wave for further analysis (Tests 5–10). The aim of this phase was to identify the frequency at which memristive behavior––specifically a pinched hysteresis loop––became apparent.

**Fig 5:**
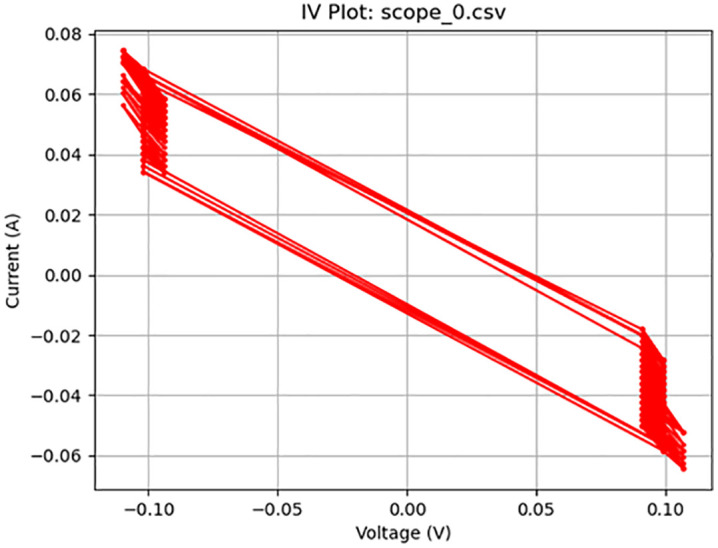
Memcapacitive activity. Plot of a 200 mV_pp_ square wave at 200 Hz displaying memcapacitive behavior.

**Fig 6:**
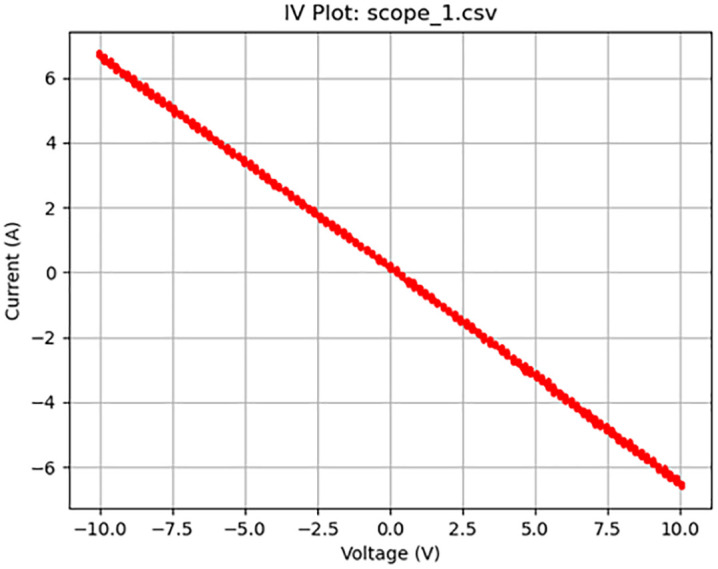
Resistive activity. Plot of a 20 V_pp_ square wave at 200 Hz displaying resistive behavior.

**Fig 7:**
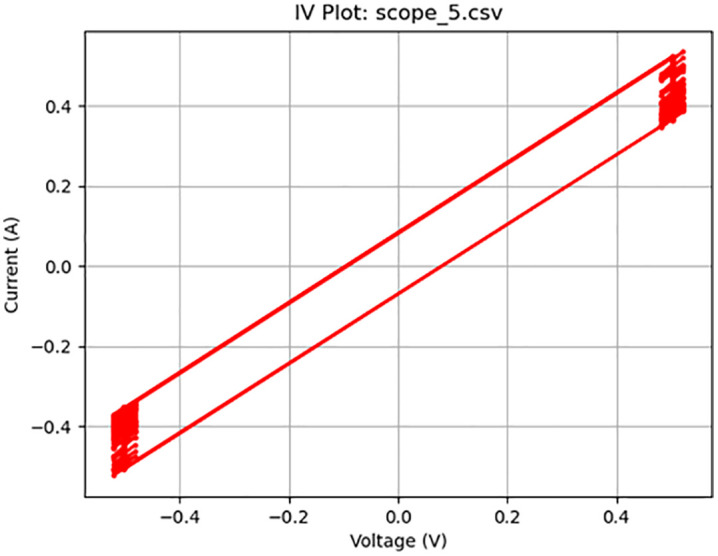
Memcapacitive activity at 200 Hz. Plot of a 1 V_pp_ square wave at 200 Hz displaying memcapacitive behavior.

**Fig 8:**
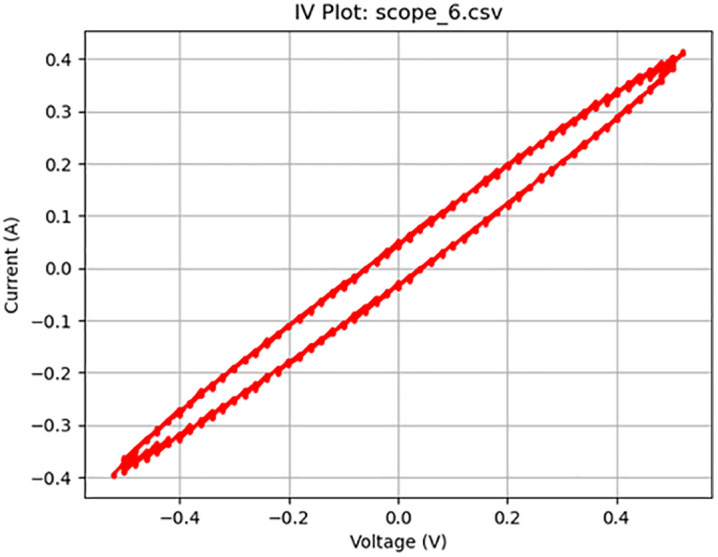
Further memcapacity activity at 200 Hz. Plot of a 1 V_pp_ sine wave at 200 Hz displaying memcapacitive behavior.

**Fig 9:**
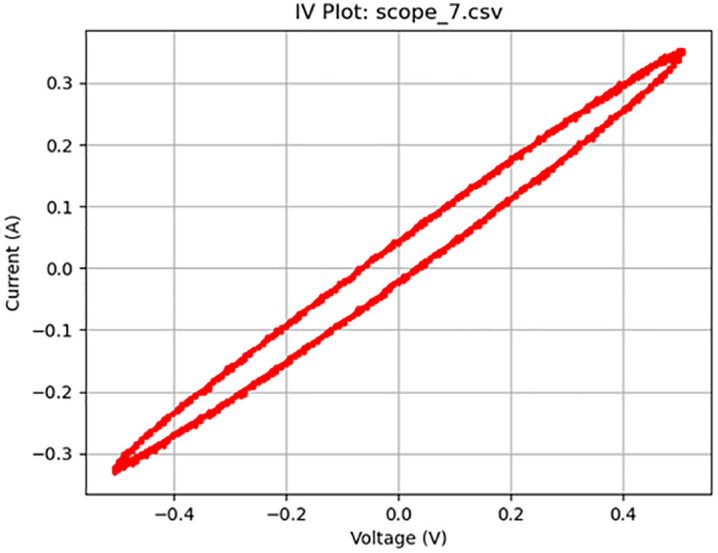
Memcapacitive activity at 100 Hz. Plot of a 1 V_pp_ sine wave at 100 Hz displaying memcapacitive behavior.

**Fig 10:**
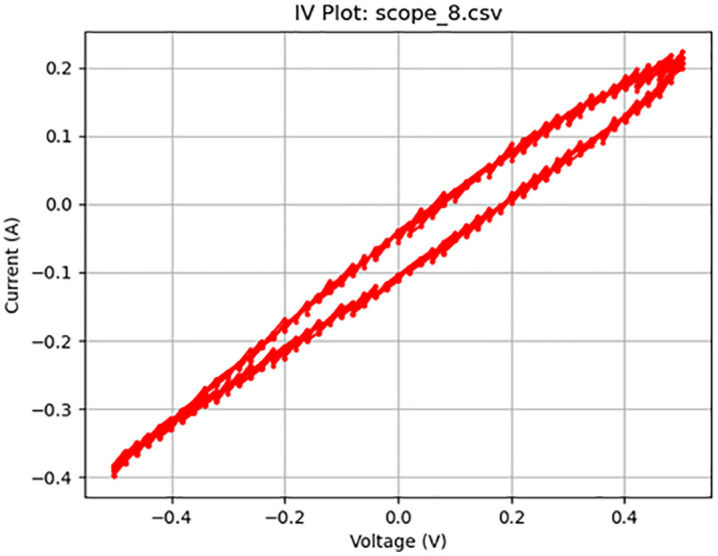
Memristive activity at 25 Hz. Plot of a 1 V_pp_ sine wave at 25 Hz displaying memristive behavior.

**Fig 11:**
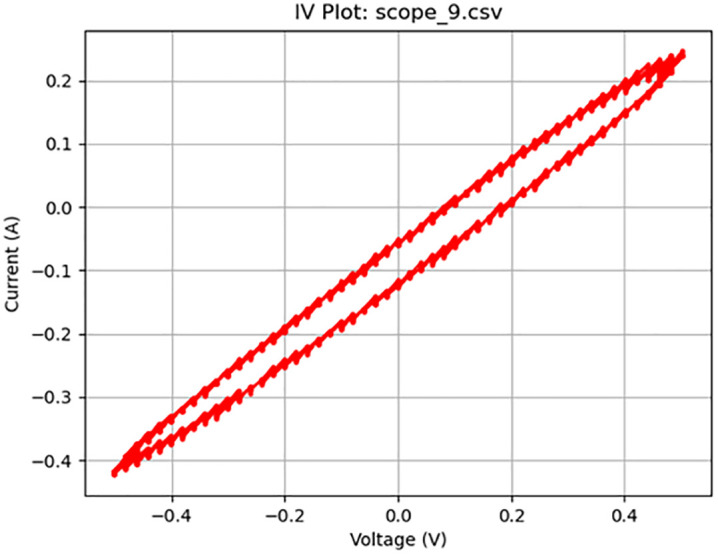
Memristive activity at 50 Hz. Plot of a 1 V_pp_ sine wave at 50 Hz displaying memristive behavior.

**Fig 12:**
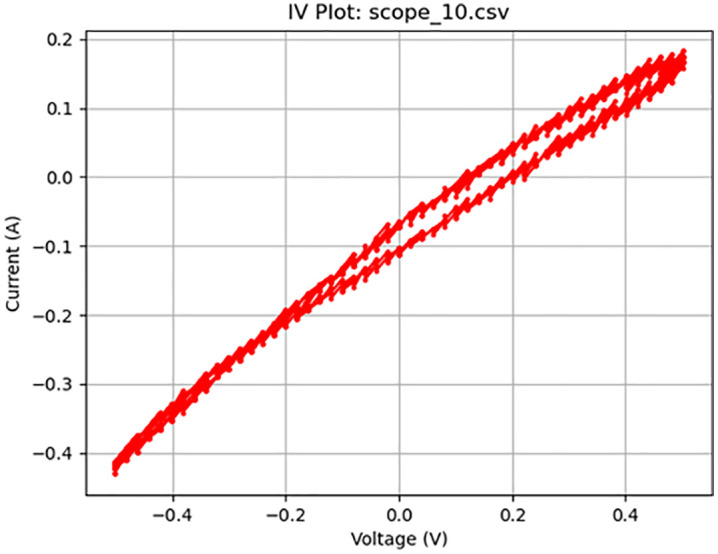
Increasingly ideal memristive activity at 50 Hz. Plot of a 1 V_pp_ sine wave at 10 Hz displaying memristive behavior.

**Fig 13:**
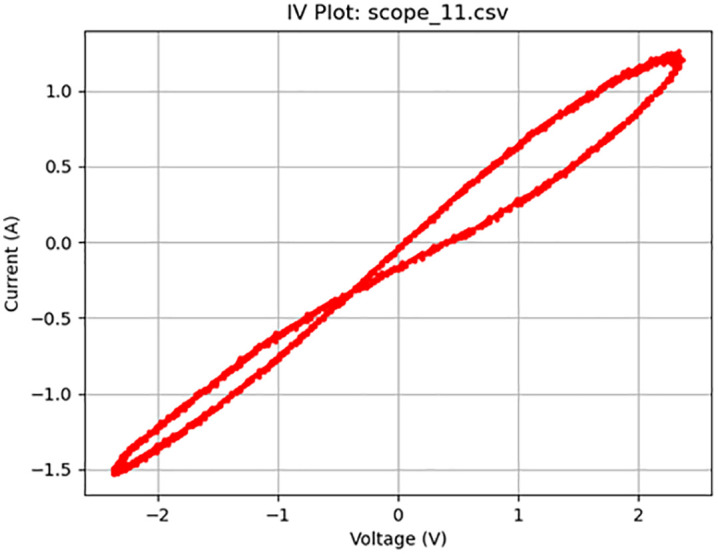
Near ideal memristive behavior. Plot of a 5 V_pp_ sine wave at 10 Hz displaying near-ideal memristive behavior.

**Fig 14:**
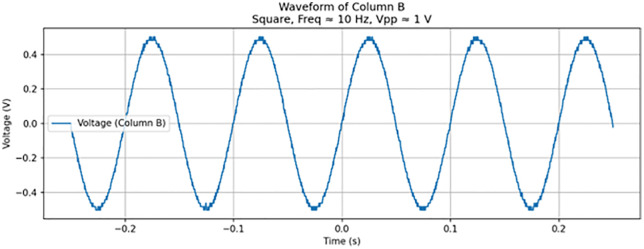
Noise profile from measurement from Test 9. Plot of a noisy 1 V_pp_ sine wave at 10 Hz during measurement.

**Fig 15:**
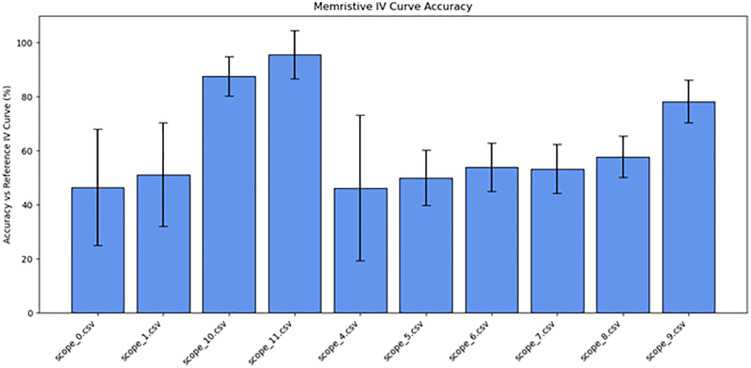
Memristive accuracy of initial tests. Memristive accuracy plotted for Tests 1-11.

In Tests 1–5, the voltage amplitude was optimized using square waves. Between Tests 5 and 6, the waveform was changed from square to sine. From Tests 6–10, frequency sweeps were carried out with sine waves to identify memristive crossing. In Test 11, the voltage range was expanded at 10 Hz (5 V_pp_) to enhance the response. This revealed behavior close to that of an ideal memristor. Notably, Test 1 had already shown consistent linear behavior, indicating resistive characteristics. The results are shown in Figs 5–13. Fig 14 details a sample noise profile, and Fig 15 summarizes memristive accuracy.

Figs 5–10 show the output of Tests 2–7. The frequency was gradually reduced until a crossing near the origin was first observed, as shown in Fig 10. To ensure this result was not an outlier caused by overshooting the ideal frequency, the test was repeated at a slightly higher frequency (50 Hz, Test 8, shown in Fig 11). This confirmed that the optimal response occurred below 25 Hz.

As summarized in Table 2, the frequency was decreased to 10 Hz (Test 9, shown in Fig 12), which produced a clear crossing in the I–V curve near the −0.4 V region. To enhance the visibility of this behavior, the voltage was increased to 5 V_pp_, which resulted in a more pronounced memristive signature (Test 10). Fig 13 illustrates this result, displaying a nearly ideal pinched hysteresis loop indicative of memristor functionality. The highest accuracy, at 95%, was at a 10 Hz sine wave at 1 V. Fig 14 details the noise from an individual sample. Fig 15 details the average memristive accuracy of each configuration.

**Table 2:** Summary of frequency, wave, voltage, behavior, and output measurements.

Figure	Test	Sampling Rate (Hz)	Waveform	Frequency (Hz)	Voltage (Vpp)	Output Name	Observed Behavior	Accuracy (%)	SE (%)
5	2	4000	Square	200	0.2	scope_0.csv	Memcapacitor	46	21
6	3	4000	Square	200	20	scope_1.csv	Resistive	51	19
7	4	4000	Square	200	1	scope_5.csv	Memcapacitor	50	10
8	5	4000	Sine	200	1	scope_6.csv	Memcapacitor	54	9
9	6	4000	Sine	100	1	scope_7.csv	Memcapacitor	53	9
10	7	4000	Sine	25	1	scope_8.csv	Memristive	58	8
11	8	4000	Sine	50	1	scope_9.csv	Memcapacitor	78	8
12	9	4000	Sine	10	1	scope_10.csv	Memristive	88	7
13	10	4000	Sine	10	5	scope_11.csv	Memristive	95	9

### Volatile memory experiment

**Fig 16:**
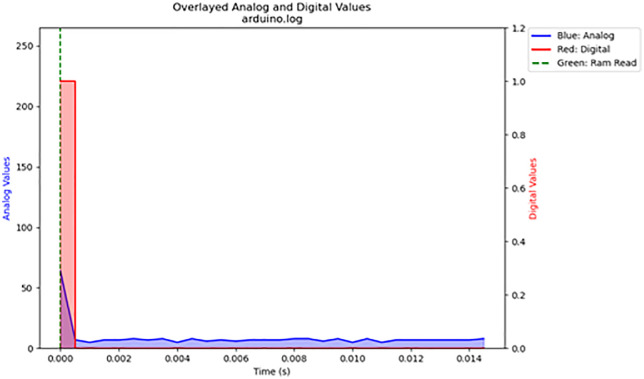
Volatile memory test 1. A single write and read over volatile memory.

**Fig 17:**
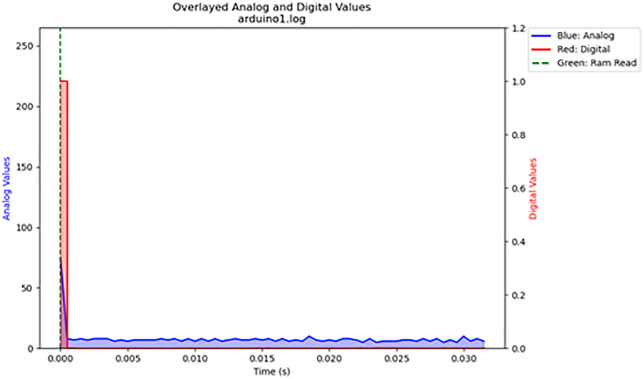
Volatile memory test 2. Another single write and read over volatile memory.

**Fig 18:**
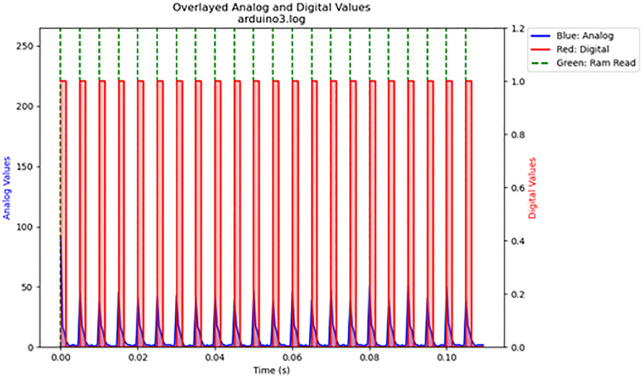
Volatile memory test 3: cyclic memory test at 200 Hz. Cyclical writing and reading over the fungal volatile memory.

**Fig 19:**
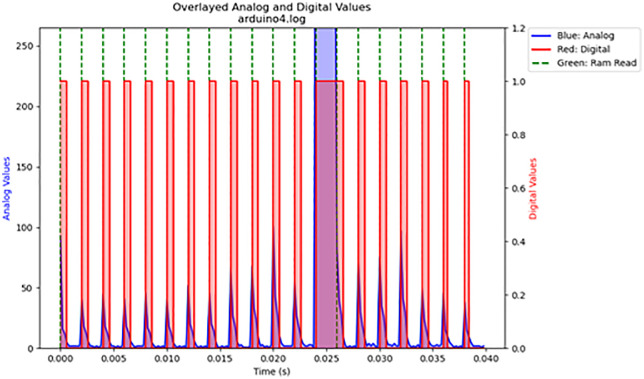
Volatile memory test 3: rapid cycle memory test at 700 Hz. Additional cyclical writing and reading over the fungal volatile memory.

**Fig 20:**
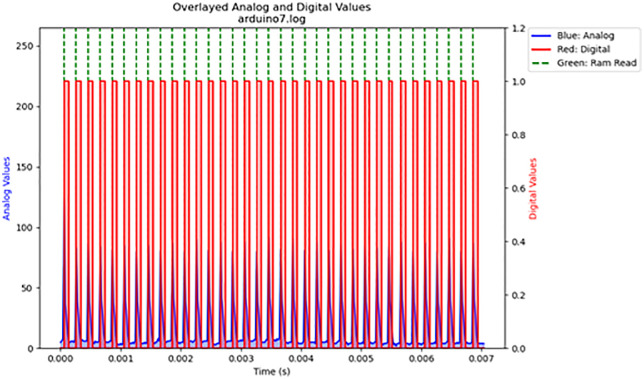
Volatile memory test 3: stressed memory test at 5.85 kHz. Extreme cyclical writing and reading from volatile memory.

**Fig 21:**
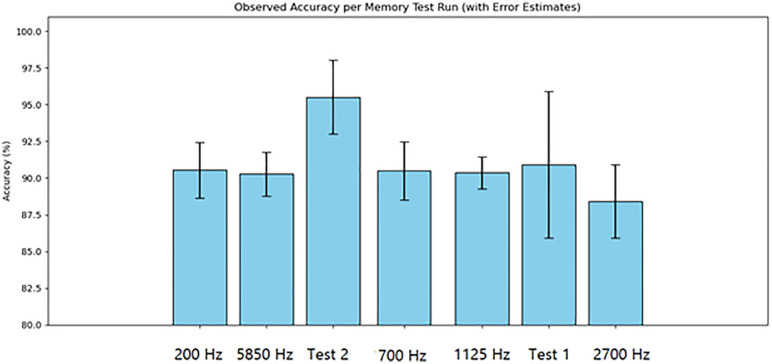
Volatile memory test memristive accuracy. Accuracy for first two tests and cyclic tests.

For the volatile memory tests 1 and 2, single read and write operations were performed across the memristor voltage divider. For volatile memory test 3, continuous read and write operations were performed across the memristor voltage divider while the frequency was gradually increased. The primary results are summarized in Table 3. The results are displayed in Figs 16–20. Averaged nemristive accuracy is displayed in Fig 21.

**Table 3:** Summary of memristive testing and measurements.

Figure	Trial	File Name	Frequency (Hz)	Accuracy (%)	SE (%)
16	1	arduino.log	<1	96	5
17	2	arduino1.log	<1	96	3
18	3	arduino3.log	200	91	2
19	3	arduino4.log	700	90	2
N/A	3	arduino5.log	1125	88	2
N/A	3	arduino6.log	2700	90	1
20	3	arduino7.log	5850	90	1

## Discussion

### Overview

Using low-cost materials, shiitake mushrooms were cultured into ideal memristors. Ideal and non-ideal memristive properties have been observed previously in fungi, but these required far more complex interfacing methods [26]. Although several techniques have been proposed to preserve fungal samples, we obtained experimental validation that dehydration can preserve the observed properties in a previously “programmed” sample [27]. Ideal memristor properties are observed at lower frequencies, but potential latencies can be offset through massive parallelization, as in nature [26,28,29]. As known from previous works on fungal memristors, the mycelial structure contains capacitive, memfractive, and memristive proteins [25,26]. In the memristive tests, accuracy decreased as the frequency increased. The observed rapid switching speed of 5,850 Hz, an accuracy of 90% (± 1%) low energy consumption relative to prior conventional systems, light weight, and radiation resistance all make fungal memristors attractive for edge computing, aerospace, and embedded firmware applications [25–27]. Unlike expensive conventional memristors, culturing fungal memristors does not require large facilities or rare minerals. The process can be scaled to grow large systems, which can be programmed and preserved for long-term use at low cost.

### Limitations

Our study was limited by the relatively short timescale of less than two months. Other researchers have documented memristive properties in mycelial materials, but their studies also focused on short-term performance [26]. Another limitation was that only single, relatively bulky samples were prepared. To truly compete with conventional devices at the microscale and below, memristors will need to be far smaller [7,8,11]. Even in the same growth medium, each sample produced a vastly different culture, and the outcomes have yet to be fully characterized by electrical properties. However, the development of these devices is in an early stage, and they could eventually be miniaturized, especially using improved cultivation techniques. Complications associated with the growth media were not explored, although previous research has found that fungi are quite robust to varying conditions [26].

### Future work

Although fungal memristors can be produced at low cost, certain aspects of the process could be further optimized. First, consistent cultivation techniques could be improved using three-dimensional (3D)-printed templates and structures that shape the shiitake mushroom into the desired geometry. Second, programming could be facilitated by adding electrical contacts into a 3D-printed cultivation structure. Finally, long-term use would necessitate preservation, which could involve a variety of techniques, including dehydration, desiccation, freeze-drying, certain hydrogels, and special coatings [27]. By testing devices produced to physical stress conditions, a combination of these techniques could enable the development of fast, radiation-resistant, and low-energy memristors grown from low-cost organic materials. The future of computing could be fungal.

## Conclusions

Currently, the fabrication of semiconductor memristors requires rare-earth minerals and large facilities, and culturing delicate neural organoids requires a complex chemical environment to be maintained in a bioreactor. Fungal computing may provide a robust and accessible alternative. Fungal systems have lower power requirements, lighter weights, faster switching speeds, and lower industrial overheads than conventional devices. In this study, fungal memristors were fabricated, programmed, and tested using shiitake mushrooms and conventional electronics. Dehydration-based preservation was successfully explored, demonstrating the robustness of our devices. When used as RAM, our mushroom memristor was able to operate at up to 5,850 Hz at an accuracy of 90 ± 1%. In addition, shiitake mushrooms are biodegradable and have demonstrated radiation resistance, suggesting that the potential applications of fungal computing range from sustainable computing devices to aerospace technologies.
